# Simultaneous outbreaks of respiratory disease in wild chimpanzees caused by distinct viruses of human origin

**DOI:** 10.1080/22221751.2018.1563456

**Published:** 2019-01-21

**Authors:** Jacob D. Negrey, Rachna B. Reddy, Erik J. Scully, Sarah Phillips-Garcia, Leah A. Owens, Kevin E. Langergraber, John C. Mitani, Melissa Emery Thompson, Richard W. Wrangham, Martin N. Muller, Emily Otali, Zarin Machanda, David Hyeroba, Kristine A. Grindle, Tressa E. Pappas, Ann C. Palmenberg, James E. Gern, Tony L. Goldberg

**Affiliations:** a Boston University, Boston, MA, USA; b University of Michigan, Ann Arbor, MI, USA; c Harvard University, Cambridge, MA, USA; d University of New Mexico, Albuquerque, NM, USA; e University of Wisconsin–Madison, Madison, WI, USA; f Arizona State University, Tempe, AZ, USA; g Makerere University, Kampala, Uganda; h Tufts University, Medford, MA, USA

**Keywords:** *Pneumoviridae*, *Metapneumovirus*, *Paramyxoviridae*, *Respirovirus*, Human respirovirus 3, Parainfluenza virus 3, Anthroponoses, Zoonoses, chimpanzee, Africa, Uganda, respiratory disease, Epidemiology, outbreak

## Abstract

Respiratory viruses of human origin infect wild apes across Africa, sometimes lethally. Here we report simultaneous outbreaks of two distinct human respiratory viruses, human metapneumovirus (MPV; *Pneumoviridae*: *Metapneumovirus*) and human respirovirus 3 (HRV3; *Paramyxoviridae*; *Respirovirus*, formerly known as parainfluenza virus 3), in two chimpanzee (*Pan troglodytes schweinfurthii*) communities in the same forest in Uganda in December 2016 and January 2017. The viruses were absent before the outbreaks, but each was present in ill chimpanzees from one community during the outbreak period. Clinical signs and gross pathologic changes in affected chimpanzees closely mirrored symptoms and pathology commonly observed in humans for each virus. Epidemiologic modelling showed that MPV and HRV3 were similarly transmissible (*R*_0_ of 1.27 and 1.48, respectively), but MPV caused 12.2% mortality mainly in infants and older chimpanzees, whereas HRV3 caused no direct mortality. These results are consistent with the higher virulence of MPV than HRV3 in humans, although both MPV and HRV3 cause a significant global disease burden. Both viruses clustered phylogenetically within groups of known human variants, with MPV closely related to a lethal 2009 variant from mountain gorillas (*Gorilla beringei beringei*), suggesting two independent and simultaneous reverse zoonotic origins, either directly from humans or via intermediary hosts. These findings expand our knowledge of human origin viruses threatening wild chimpanzees and suggest that such viruses might be differentiated by their comparative epidemiological dynamics and pathogenicity in wild apes. Our results also caution against assuming common causation in coincident outbreaks.

## Introduction

Respiratory viruses of human origin have caused disease in wild apes across Sub-Saharan Africa and pose a significant and growing threat to wild ape health and conservation [1,2]. For example, respiratory disease is the leading cause of morbidity and mortality among chimpanzees (*Pan troglodytes*) in Gombe Stream National Park, Tanzania [3,4] and in Kibale National Park, Uganda [5], two populations that have been studied continuously for decades. Mortality from anthroponotic respiratory pneumoviruses (family *Pneumoviridae*) and paramyxoviruses (family *Paramyxoviridae*) has been documented in western chimpanzees (*P. t. verus*) in Cote d'Ivoire [1,6], eastern chimpanzees (*P. t. schweinfurthii*) in Tanzania [7], mountain gorillas (*Gorilla beringei beringei*) in Rwanda, lowland gorillas (*G. g. gorilla*) in Central African Republic [8] and bonobos (*P. paniscus*) in the Democratic Republic of the Congo [9]. Rhinovirus C [10] and coronavirus OC43 [11] of human origin have also caused chimpanzee mortality in Uganda and mild respiratory disease in Cote d'Ivoire, respectively.

Biological similarities between humans and apes predispose them to cross-species pathogen transmission [12], and habitat alterations may exacerbate inter-species contact and anthroponotic transmission risk [2]. Although simultaneous infections of apes and people with the same respiratory virus have rarely been confirmed directly [8,11], viruses that are relatively benign in humans can cause lethal outbreaks in ape populations, indicating lack of resistance in apes. Suitable prevention strategies have included improved hygiene and sanitation [13], reduced human visitation [14] and large-scale vaccination of apes (if effective vaccines someday become available) [15]. Such policies could also benefit human public health by reducing zoonotic transmission risk [16,17].

Here, we report simultaneous outbreaks of respiratory disease in two nearby chimpanzee communities in Uganda, caused by two distinct negative-sense RNA viruses of human origin. The outbreaks occurred from December 2016 to February 2017 in the Ngogo and Kanyawara chimpanzee communities in Kibale National Park [18] (Figure S1). Only 10 km apart, the communities are interconnected by contiguous moist evergreen forest, separated by only one intervening chimpanzee community that is not currently studied, and both communities generally experience low mortality rates [19,20].

Although outbreaks of respiratory disease in wild chimpanzees are common, their causes often remain undiagnosed [10]. Fresh carcasses are often not recovered, and the remoteness of field sites complicates sample storage and analysis. Non-invasive diagnostics (usually from feces) have proven highly informative [6], but negative results in such cases are inconclusive [10]. In the present case, rapid recovery of carcasses, the presence of trained veterinarians and the availability of basic field laboratory capabilities on site provided an opportunity for etiologic diagnoses. Furthermore, coordinated, prospective collection of observational data between the two sites offered an unusual chance to compare the clinical and epidemiologic features of the outbreaks directly.

## Results

**Figure 1.**
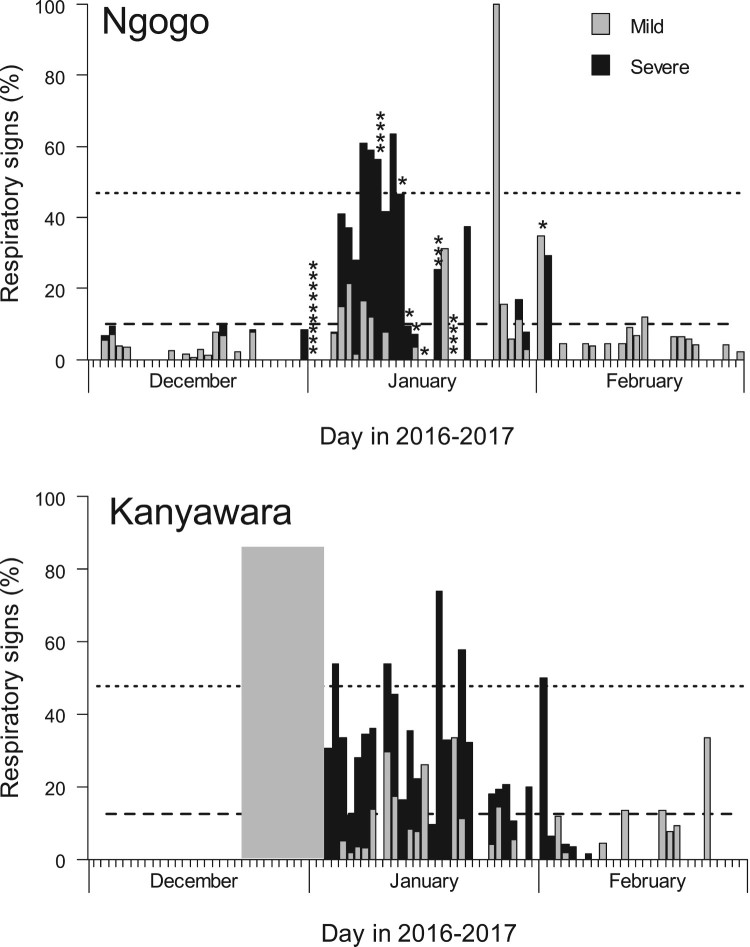
Epidemic curves for the 2016/2017 chimpanzee respiratory disease outbreaks at Ngogo (top) and Kanyawara (bottom) communities in Kibale National Park, Uganda. Dotted lines indicate 2017 mean rates of respiratory signs (lower line) and 2 standard deviations above the mean (upper line). Asterisks above bars indicate the estimated timing of individual mortality events attributed to respiratory disease. The grey box in the lower graph represents dates with missing data (no clinical signs were observed at Kanyawara before this period).

From 31 December 2016 to 8 February 2017, the Ngogo chimpanzee community of Kibale National Park, Uganda (Figure S1), experienced an outbreak of severe respiratory disease. During the same period, the nearby Kanyawara community (Figure S1) also experienced a respiratory disease outbreak. At the onset of the epidemics, Ngogo community consisted of 205 chimpanzees from <1 year to 67 years old, and Kanyawara community consisted of 55 chimpanzees from <1 year to 51 years old. Epidemic curves (Figure 1) show that the Ngogo and Kanyawara outbreaks each occurred in a single phase, with most cases occurring in January 2017. At Ngogo, 43.8% of chimpanzees observed between 3 December 2016 and 28 February 2017 exhibited respiratory signs. At Kanyawara, 69.1% of chimpanzees observed during the same period exhibited respiratory signs. At Ngogo, 25 chimpanzees (12.2%) died during the outbreak period (Figure 1). In contrast, no chimpanzees at Kanyawara died during the outbreak period, with the exception of a female recovering from disease who died following conspecific aggression (see below). Respiratory signs consisted of coughing, sneezing, dyspnea, and nasal exudate; other signs included lethargy, immobility, and dramatic loss of body condition (Figure S2).

Epidemiologic modelling of the Ngogo and Kanyawara outbreaks (Table 1 and Figure S3) yielded daily transmission rate estimates of 1.13 and 0.338, and durations of infectivity of 1.12 and 4.55 days, respectively. These parameters yielded basic reproductive numbers (*R*_0_) of 1.27 and 1.48 for Ngogo and Kanyawara, respectively. These are similar to values estimated from an outbreak of rhinovirus C in Kanyawara in 2013 (Table 1) during which 8.9% of chimpanzees died, and to published values for the human “common cold” [10]. However, 95% confidence limits around these estimates were non-overlapping for daily transmission rates of all three outbreaks and duration of infectivity and *R*_0_ in Ngogo (both lower than in Kanyawara in 2013 or 2016/2017).

**Table 1.** Epidemiologic parameters derived from SIR mathematical models of the metapneumovirus and human respirovirus 3 outbreaks in chimpanzees from Ngogo and Kanyawara communities, respectively. Similarly derived parameters from a 2013 outbreak of human rhinovirus C (Kanyawara only) are shown for comparison (95% confidence intervals in parentheses) [10].

	Ngogo 2017	Kanyawara 2017	Kanyawara 2013
Causative agent	Metapneumovirus	Human respirovirus 3	Rhinovirus C
Epidemic size	78 individuals	38 individuals	31.7 individuals
Epidemic duration	35 days	>33 days	20.7 days
Mortality	25 individuals	0 individuals	5 individuals
Transmission rate (*β*)	1.13/day (1.12–1.15)	0.326/day (0.312–0.340)	0.68/day (0.44–0.85)
Duration of infectivity (1/*γ*)	1.12 days (1.10–1.13)	4.55 days (4.30–4.83)	3.2 days (1.6–5.8)
*R*_0_ (*β*/*γ*)	1.27 (1.23–1.30)	1.48 (1.34–1.64)	1.83 (1.38–2.56)

Risk factor analysis (Table S2) showed that age significantly predicted morbidity at both Ngogo (χ^2^ = 10.097, DF = 3, *p* = 0.018) and Kanyawara (χ^2^ = 12.154, DF = 3, *p* = 0.007). In both communities, respiratory signs were least frequently observed among infants and increased through successive age categories. Sex did not affect morbidity or mortality at Ngogo, but females were significantly more likely to exhibit respiratory signs at Kanyawara than were males (χ^2^ = 6.310, DF = 1, *p* = 0.012). At Ngogo, age predicted mortality (χ^2^ = 19.153, DF = 3, *p* < 0.001), with mortality highest among infants (OR 5.01, 95% CI: 1.53–19.56) and individuals ≥30 years old (OR 3.86, 95% CI: 1.16–15.15), compared to intermediate ages.

At Ngogo, the carcass of a 20-year-old female chimpanzee was recovered immediately after the onset of respiratory signs and subsequent death. Post-mortem analysis of this individual revealed consolidation of the dependent lobes of both lungs and a serosanguinous pericardial effusion but no other gross pathologic abnormalities (Figure S4). At Kanyawara, the carcass of a 22-year-old female chimpanzee was recovered approximately 10 days after having recovered from respiratory signs (but still remaining weak), immediately after having been attacked by conspecifics (for unclear reasons). Post-mortem analysis of this individual showed severe, diffuse pleuropneumonia with fibrinous adhesions to the thoracic wall and severe consolidation of all lobes of both lungs, as well as a serosanguinous pericardial effusion (Figure S4).

Analysis of paired fecal samples (prior to and during the outbreak period) using a Luminex assay that tests for a suite of human respiratory agents revealed different viral etiologies for each community (Table 2). Metapneumovirus (MPV, *Pneumoviridae*: *Metapneumovirus*) was detected in 7 of 11 individuals (63.6%) from Ngogo chimpanzees exhibiting clinical signs during, but not before, the outbreak period (Fisher’s exact *P* = 0.0030). Human respirovirus 3 (HRV3; *Paramyxoviridae*; *Respirovirus*, formerly known as parainfluenza virus 3) was detected in 5 of 14 individuals (35.7%) from Kanyawara chimpanzees exhibiting clinical signs during, but not before, the outbreak period (Fisher’s exact *P* = 0.0005). Adenoviruses (*Adenoviridae*) were present in samples from both Ngogo (36.4%) and Kanyawara (78.6%) but showed no association with the outbreak period (Fisher’s exact *P* = 0.4545 and 0.5291, respectively) and have been previously characterized in this population at comparable frequencies [10]. Enteroviruses (*Picornaviridae*) were also present at low frequency in samples from both communities, similarly showed no association with the outbreak period (Fisher’s exact *P* = 1.000 in both cases), and have also been previously characterized in this population at comparable frequencies [10].

**Table 2.** Results of diagnostic testing by Luminex assay of paired fecal samples from chimpanzees in Ngogo and Kanyawara communities before (Q4, 2016) and during (Q1, 2017) the outbreak period, respectively.

Ngogo												
		AdV			EV			MPV			HRV3	
*ID*		Q4/16	Q1/17		Q4/16	Q1/17		Q4/16	Q1/17		Q4/16	Q1/17
* *												
AB												
BA												
BT												
CN												
DX												
GT												
MI												
PT												
WI												
WN												
ZL												
												
*Kanyawara*												
		AdV			EV			MPV			HRV3	
*ID*		Q4/16	Q1/17		Q4/16	Q1/17		Q4/16	Q1/17		Q4/16	Q1/17
* *												
AL												
AN												
AT												
AZ												
BT												
LK												
OG												
PO												
QV												
RD												
UM												
WC												
WL												
WO												
												

Viruses are adenovirus (AdV), enterovirus (EV), metapneumovirus (MPV) and human respirovirus 3 (HRV3). Shaded cells indicate positive results for the individuals listed by two-letter abbreviation in the first column, all of whom became clinically ill during the outbreak period. Results for other viral and bacterial pathogens included in the assay were negative.

Metagenomic analysis of respiratory tract swab samples from the chimpanzee examined postmortem at Ngogo yielded 16,107,924 reads after trimming, of which 27,393 assembled to yield a coding-complete MPV genome of 13,230 bases with average coverage of 196, consistent with the Luminex results described above. This genome (GenBank accession number MH428626) was most similar (98.69%) to a 2010 human-derived variant from Brazil (GenBank accession number MG431250). Intriguingly, the virus was nearly as similar (98.67%) to a variant from a mountain gorilla from Rwanda in 2008 (GenBank accession number HM197719) detected during a lethal outbreak [21]. MPV RNA was present in all sections of the respiratory tract, including the lung parenchyma, with the proportion of viral sequence reads declining monotonically from the upper to the lower respiratory tract (Figure S5). Sequencing of a 480 nucleotide portion of the viral F gene from fecal samples was successful for three other Luminex-positive chimpanzees at Ngogo (AB, MI and WI; Table 2), yielding identical sequences within this variable genomic region (GenBank accession numbers MH428628- MH428630).

By contrast, neither HRV3 nor any other virus was detected in the respiratory tract of the chimpanzee that died at Kanyawara, likely reflecting prior infection and viral clearance. A coding-complete HRV3 genome (15,407 bases) was therefore reconstructed from a fecal sample from this same individual collected when she was coughing approximately 2 weeks earlier, using PCR with virus-specific primers and Sanger sequencing (Table S1). This genome (GenBank accession number MH428627) was most similar (99.38%) to a 2009 human-derived variant from the USA (GenBank accession number KY674929). Sequencing of a variable and epidemiologically informative 348-nucleotide portion of the viral F gene from fecal samples was successful for three other Luminex-positive chimpanzees at Kanyawara (AL, AN and AZ; Table 2), yielding identical sequences within this genomic region (GenBank accession numbers MH428631–MH428633). Re-analysis of respiratory tract metagenomic data from this individual and the individual from Ngogo revealed that a small proportion of reads in the upper respiratory tracts of both animals (0.05% and 0.16%, respectively) mapped to the reference genome of *Staphylococcus pneumoniae* (GenBank accession number NC_003098), which can infect chimpanzees secondarily during viral respiratory disease outbreaks [9,22], indicating the presence of this or a related bacterium; however no reads mapped to this organism in the lungs of either animal.

**Figure 2.**
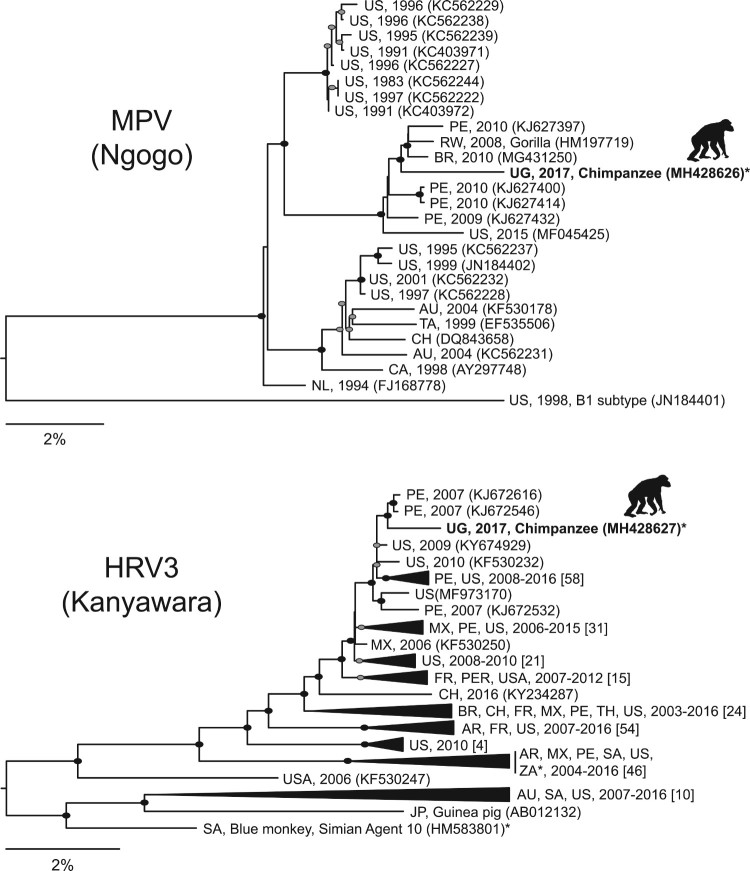
Maximum likelihood phylogenetic trees of metapneumovirus (MPV) from Ngogo (top) and human respirovirus 3 (HRV3) from Kibale (bottom) constructed from nucleotide alignments of coding-complete viral genomes. Taxon names indicate country of origin (AU = Australia; BR = Brazil; CA = Canada; CH = Chile; FR = France; J*P* = Japan; MX = Mexico; NL = Netherlands; PE = Peru; RW = Rwanda; SA = South Africa; TA = Taiwan; TH = Thailand; UG = Uganda; US = USA; ZA = Zambia), year of collection (if specified), and GenBank accession number in parentheses. For HRV3, major clades were collapsed for visualization, and numbers of sequences within each clade are shown in brackets. Sequences generated in this study from chimpanzees are in bold with silhouettes above. Filled ellipses indicate bootstrap values of 100%; grey ellipses indicate bootstrap values ≥75%. Scale bars indicate nucleotide substitutions per site. Asterisks indicate sequences from other non-human primates (see text for details). Full details of all sequences included are in given in Table S3.

Phylogenetic analysis revealed MPV from the Ngogo outbreak to sort within a sub-clade of subtype B2 [23] viruses from Brazil, Peru, Rwanda and the USA (Figure 2). This sub-clade contains recently collected viruses (2009–2015), including the mountain gorilla variant from Rwanda. The MPV variant from Ngogo belongs to a different subtype than a previously reported B1 virus from a chimpanzee outbreak in Tanzania [7], and it is less closely related to a previously reported B2 subtype from a chimpanzee outbreak in Cote d'Ivoire [1] than to other variants within its subclade, including the gorilla-derived variant, based on the available 867 nucleotide region of the viral *P* gene (GenBank accession numbers EU240454-EU240456; not shown).

Phylogenetic analysis revealed HRV3 from the Kanyawara outbreak to sort within a sub-clade of highly similar human-derived viruses from Peru and the USA collected between 2006 and 2015 (no more recently than other sub-clades; Figure 2). Notably, HRV3 from Kanyawara was divergent from viruses from other non-human primates, including viruses from wild Zambian baboons (*Papio cynocephalus*) [24] and simian agent 10, originally isolated from a blue monkey (*Cercopithecus mitis*) in South Africa [25].

## Discussion

Outbreaks of severe and sometimes lethal respiratory disease are common in chimpanzees across Sub-Saharan Africa, but their causes often remain undetermined [2,10]. Consequently, and because of differences in field protocols among research sites, it has proven difficult to compare these outbreaks directly. The simultaneity of the two 2016/2017 outbreaks at Ngogo and Kanyawara, coupled with coordinated data and sample collection efforts between the two sites, allowed a rare direct comparison to be made. Analyses demonstrate that the two viruses displayed different pathogenicities and different epidemiological dynamics in the two chimpanzee communities.

MPV at Ngogo caused substantially higher mortality than HRV3 at Kanyawara, despite causing apparently lower morbidity. However, the estimated daily transmission rate and duration of infectivity of MPV at Ngogo differed from corresponding values for HRV3 at Kanyawara (Table 1), suggesting that, despite comparable *R*_0_ values, the viruses displayed different epidemiologic dynamics. Higher mortality due to MPV in infants and older adults compared to intermediate age categories is congruent with data from humans, where infants, young children and older adults are most susceptible to severe clinical manifestations of MPV infection [26,27], as well as with demographic data from prior MPV outbreaks in chimpanzees [1,6].

Neither virus was detected in chimpanzees immediately prior to the outbreaks (Table 2). MPV was detected in 63.6% of affected Ngogo chimpanzees sampled during the outbreak, and HRV3 was detected in 35.7% of affected Kanyawara chimpanzees sampled during the outbreak. Viral sequences from affected individuals were identical within the variable region sequenced. MPV and HRV3 were therefore likely single-origin causes of their respective epidemics. Adenoviruses and enteroviruses were present both before and during the outbreaks at statistically indistinguishable (AdV) and low (EV) frequencies. Adenoviruses occur in both healthy and ill wild apes [28–31] and in chimpanzees during respiratory outbreaks [32], including at Kanyawara [10], leading to debate about their etiologic role; however, our results support previous conclusions that these viruses are most likely benign. Our results support a similar conclusion for enteroviruses, with the notable exceptions of polio virus [3,33] and rhinoviruses [10], which can cause deadly disease in chimpanzees.

We note that, at the time of the outbreaks, the Ngogo and Kanyawara epidemics were assumed to have the same cause, with concomitant fears that an infectious agent was spreading rapidly across Kibale National Park. Moreover, avian influenza had recently been reported in waterfowl in Lake Victoria, on the other side of Uganda [34]. Our results highlight how heightened vigilance for emerging infectious diseases can sometimes lead to erroneous inferences. Common environmental drivers (e.g. weather, human visitation patterns) may precipitate simultaneous outbreaks of distinct anthroponotic viruses in wild apes, creating the appearance of a single disease with a seasonal pattern. Our results also suggest that the transmission of respiratory viruses between chimpanzee communities is probably uncommon, despite high rates of transmission within chimpanzee communities. Chimpanzees from neighbouring communities rarely interact [33], which may limit opportunities for inter-community transmission even of highly contagious microbes.

MPV and HRV3 both represent significant burdens to global public health [35]. MPV infection in humans causes coughing, nasal discharge and weight loss [36]; in non-human models signs are often more severe than for other related viruses [37]. MPV also causes clinical disease in experimentally infected chimpanzees [38] and severe and sometimes lethal outbreaks of respiratory disease in wild chimpanzees and gorillas, often with similar demographic patterns of infection as we describe herein [1,6,7,21], as does a related virus, respiratory syncytial virus (*Pneumoviridae*, *Orthopneumovirus*) [1]. Our results show that this comparatively high virulence of MPV is recapitulated in naturally infected chimpanzees.

HRV3 causes respiratory disease and asthma exacerbation in people worldwide, especially in children and the elderly, but it is less virulent than MPV [39]. HRV3 has a wide host range, including primates, ruminants and rodents [40]. Simian agent 10, first isolated in 1963 from an apparently healthy blue monkey [41], was later identified as HRV3 and attributed to anthroponotic transmission [25]. HRV3 has been detected in the respiratory tracts of apparently healthy wild yellow baboons in Zambia [24]. Experimental infection of marmosets (*Saguinus mystax*) with HRV3 causes acute and transmissible disease [42], but experimental infection of other primates, including rhesus macaques (*Macaca mulatta*) and chimpanzees [43], is milder. Our results mirror these findings and, more generally, show that some negative-sense RNA respiratory viruses of human origin cause only mild and self-limiting infections in wild chimpanzees.

We cannot exclude the role of other co-infecting pathogens in modulating the pathogenicity of MPV and HRV3 in our study populations. For example, HRV3 can cause upper respiratory disease and predispose chimpanzees to invasive pneumococcal infection [44], and the bacterium *Streptococcus pneumoniae* co-occurs with human metapneumoviruses and respiratory syncytial viruses in both wild and in captive apes [9,22]. Our finding of a low percentage of metagenomic sequence reads matching *S. pneumoniae* (or a related bacterium) in the upper respiratory tracts of both necropsied individuals, but not in their lungs, supports the notion that wild chimpanzees host microbes that could easily invade the lower respiratory tract following primary viral infection. Population surveys of endemic microbes and serologic assessments of prior exposure to agents that might predispose chimpanzees to infection or enhance pathogenesis could prove informative.

Clinical signs in Ngogo chimpanzees were congruent with those in severely infected humans [36]. One adult female at Ngogo (“Kidman”) has exhibited persistent wheezing since the outbreak; in human children suffering severe MPV infections, subsequent wheezing episodes are not uncommon [45]. Post-mortem analysis of one individual at Ngogo revealed surprisingly little gross pathology of the respiratory tract, consistent with acute infection and rapid death, and MPV was recovered from all sections of that individual’s respiratory tract, including the lung parenchyma (Figures S4 and S5). In contrast, post-mortem analysis of an individual at Kanyawara revealed advanced respiratory tract pathology, consistent with longer term infection (Figure S5), but we did not recover HRV3 from this individual’s respiratory tract despite recovering a complete HRV3 genome from this same individual’s feces collected approximately 2 weeks earlier. This individual had likely recovered from the initial viral challenge, cleared the virus, and perhaps acquired a secondary bacterial infection [9,22]. Notably, both individuals had pericardial effusions, which in humans are rare but serious sequellae of certain viral infections [46].

Phylogenetic analyses revealed that MPV from Ngogo and HRV3 from Kanyawara are each closely related to globally circulating human viruses, indicating independent anthroponotic sources for both viruses. Apparent geographic associations in these phylogenies likely represent bias in available sequence data (e.g. high representation of viruses from Peru and the USA; Table S3). The Ngogo MPV variant clusters within a sub-clade of viruses that is more recent (2008–2017) than other sub-clades, perhaps reflecting antigenic replacement of MPV strains over time. Notably, the Ngogo variant is very similar to a virus from a fatal infection of a mountain gorilla in Rwanda in 2009 [21]. This particular viral lineage may thus have a propensity for infecting apes, or it may simply circulate widely in East Africa. The virus is divergent, however, from previously described, lethal viruses from wild chimpanzees in Tanzania [7] and Cote d'Ivoire [1], indicating that multiple MPV variants can infect and kill wild chimpanzees.

The pathways by which these viruses entered chimpanzees from humans remain vexingly obscure. People frequent the habitats of both chimpanzee communities for research and associated activities (not solely focused on chimpanzees) and to travel or collect forest products [47]. Tourism is sometimes cited as a risk for anthroponotic transmission of viruses to great apes and helps inform current International Union for the Conservation of Nature (IUCN) recommendations for preventing such transmission, such as visitor vaccination requirements, minimum observation distances, alcohol-based sanitizing gel and facemasks [13]. However, neither Ngogo nor Kanyawara is a tourism site. Ngogo is bordered by a tourism site (Kanyanchu) receiving thousands of visitors each year, but there were no reports of a concurrent respiratory disease outbreak in the Kanyanchu chimpanzees, although similar events have occurred there at other times. At Gombe National Park in Tanzania, provisioning chimpanzees with bananas (a discontinued practice) was, along with season, the strongest predictor of chimpanzee respiratory signs [48], which affected between 0% and 9% of chimpanzees per month between 2005 and 2012 [4]. However, neither the Ngogo nor the Kanyawara chimpanzees are provisioned, and biosecurity measures based on IUCN recommendations [13] for visitors to both sites are in place. At Kanyawara, data over 22 years show a seasonal pattern of respiratory illness in chimpanzees, typically peaking in March, but no correlation between clinical signs in chimpanzees and seasonal patterns of respiratory illness in humans living nearby [5]. The role of other species in transmitting these viruses from humans to chimpanzees is also unclear. Kibale National Park contains a diversity of primate species and social groups, none of which were observed with respiratory signs at the time of the outbreaks, but complex patterns of cross-species transmission from humans to chimpanzees cannot be discounted. Such possibilities could be investigated through broad sampling of people (especially research staff) and wildlife during future outbreaks, including serologic assessments of prior exposure.

Overall, these findings broaden our understanding of human viruses that can cause disease in wild chimpanzees and show that their clinical manifestations can vary markedly. Our study also shows that epidemiological analyses, enabled by prospective, coordinated observational data collection, may be able to distinguish among viruses based on early-stage infection patterns. If so, “real-time” epidemiologic analyses would be a valuable complement to molecular diagnostic testing, in that they could help guide response strategies as epidemics progress. Epidemiologic analyses could also inform future monitoring efforts to detect and minimize the impact of anthroponotic transmission events on wild ape populations.

## Materials and methods

### Ethics statement

The study was observational and non-invasive. Protocols were approved by Institutional Animal Care and Use Committees (IACUC) of Harvard University (protocol 96-03) and University of New Mexico (protocol 14-101186-MCC) and were exempt by Boston University’s and the University of Michigan’s IACUC. Protocols followed the Weatherall Report, NIH Guide for the Care and Use of Laboratory Animals, USDA Animal Welfare Act, Institute for Laboratory Animal Research Guide for the Care and Use of Laboratory Animals, US Public Health Service and National Academies of Sciences National Research Council, and US Centers for Disease Control and Prevention.

### Observational data

During the outbreaks, trained field assistants collected individual-level observational data on the chimpanzees daily. Chimpanzees in both the Ngogo and Kanyawara communities are individually identifiable, making such data possible to collect. We compiled these data into measures of clinical signs (coughing or sneezing, further classified as mild or severe), and observation time per chimpanzee. We estimated dates of mortality events as the midpoint of the last date a chimpanzee was seen alive and the first date it was absent from a group containing frequent associates, including dependent offspring.

### Epidemiological analyses

To infer epidemiological transmission parameters, we constructed two SIR (Susceptible-Infectious-Removed) mathematical models – one for each community – following Althaus [49], fitting curves to cumulative incidence data using the Nelder and Mead optimization algorithm in the optim package in R, version 3.3.2 [50]. For both communities, we assumed free admixture and did not incorporate heterogeneity of social interaction to minimize model complexity, as we did previously for a rhinovirus C outbreak in Kanyawara [10].

We analysed risk factors for morbidity and mortality using a generalized linear model (GLM) with binomial distribution and log-link function g in R [51]. Age, sex and their interaction were included as predictor variables, and observation hours were included as a control variable. Age was modelled as a categorical variable with four levels: infants (<5 years), juveniles (5–14.9 years), adults (15–29.9 years) and older (≥30 years). At Ngogo, where mortality occurred, 13 individuals not observed with clinical signs during the outbreak period that disappeared were coded as having died and as positive for respiratory signs. Because none of these individuals were adolescent females (the only age-sex category of chimpanzee that can migrate between communities), their sudden disappearance (and the fact that they have never been seen since) confirms that they had, in fact, died.

### Molecular diagnostics

We collected fecal samples from individual chimpanzees in both communities during the outbreak period. Two millilitres of feces were placed immediately into RNAlater buffer (Thermo Fisher Scientific, Waltham, MA, USA) at a 1:1 ratio, homogenized and stored at −20°C until exported to the United States. Paired fecal samples were available from 25 clinically ill chimpanzees (11 from Ngogo and 14 from Kanyawara) during and before (fourth quarter of 2016) the outbreak period, and we tested these for a suite of respiratory pathogens using the NxTAG Respiratory Pathogen Panel (Luminex Corporation, Austin, TX, USA) as previously described [10], which tests for influenza virus A (multiple subtypes), human respiratory syncytial viruses A and B, coronaviruses (multiple subtypes), human metapneumovirus, rhinovirus/enterovirus, adenovirus, parainfluenza viruses 1–4, bocavirus, and the bacterial pathogens *Chlamydophila pneumoniae* and *Mycoplasma pneumoniae*.

Necropsies were performed on two chimpanzees recovered immediately after death (the only two carcasses recovered, despite extensive effort) by trained veterinarians wearing appropriate personal protective equipment. One chimpanzee from Ngogo died on 16 January 2017 (“Stella,” a 20-year-old female) and the other chimpanzee from Kanyawara died on January 13, 2017 (“Rwanda,” a 22-year-old female). In the latter case, the individual had been coughing 10 days earlier and was attacked repeatedly by conspecifics while still weak; the resulting trauma appeared to be the immediate cause of death. Samples (sterile swabs with plastic shafts and Dacron tips) were collected from the nose, larynx, trachea, bronchi and lung parenchyma of both individuals and stored in 0.25 ml RNAlater buffer at −20°C. Swabs were homogenized and total RNA was extracted and converted to cDNA libraries in the field, then prepared for sequencing on an Illumina MiSeq instrument (Illumina, San Diego, CA, USA) using 300 bp paired-end read chemistry as previously described [10].

### Virus genome sequencing and phylogenetic analyses

Polymerase chain reaction (PCR) and Sanger sequencing were used to complete viral genomes and to characterize viruses detected by Luminex assay in fecal samples (Table S1). To infer phylogenetic relationships among the two chimpanzee viruses and published viral sequences, we analysed full genome alignments of all available viruses in GenBank (alignment lengths 13,234 and 15,330 positions, respectively) using PhyML 3.0 [52] with 1000 bootstrap replicates of the data to assess statistical confidence in clades, and we displayed the resulting trees using FigTree 1.4.3 [53,54].

Data Availability: Data are available in GenBank under accession numbers MH428626-MH428633.

## ORCID

*Jacob D. Negrey*http://orcid.org/0000-0001-7355-0319

*Rachna B. Reddy*http://orcid.org/0000-0003-3338-2052

*Erik J. Scully*http://orcid.org/0000-0001-8823-8897

*Leah A. Owens*http://orcid.org/0000-0002-4232-3516

*John C. Mitani*http://orcid.org/0000-0001-7042-5854

*Melissa Emery Thompson*http://orcid.org/0000-0003-2451-6397

*Richard W. Wrangham*http://orcid.org/0000-0003-0435-2209

*Martin N. Muller*http://orcid.org/0000-0002-4298-8219

*Emily Otali*http://orcid.org/0000-0001-6837-1260

*Zarin Machanda*http://orcid.org/0000-0001-7060-7949

*Kristine A. Grindle*http://orcid.org/0000-0001-6557-250X

*Tressa E. Pappas*http://orcid.org/0000-0001-6706-4146

*Ann C. Palmenberg*http://orcid.org/0000-0002-9682-8480

*James E. Gern*http://orcid.org/0000-0002-6667-4708

*Tony L. Goldberg*http://orcid.org/0000-0003-3962-4913
